# A Methylene Blue-Enhanced Nanostructured Electrochemical Immunosensor for H-FABP Myocardial Injury Biomarker

**DOI:** 10.3390/bios13090873

**Published:** 2023-09-07

**Authors:** Cecília Maciel Prado, Paula Angélica Burgos Ferreira, Lucas Alves de Lima, Erika Ketlem Gomes Trindade, Rosa Fireman Dutra

**Affiliations:** Biomedical Engineering Laboratory, Department of Biomedical Engineering, Federal University of Pernambuco, Av. Prof. Moraes Rego, 1235, Recife 50670-90, Brazil

**Keywords:** immunosensor, methylene blue, polythionine, H-FABP, acute myocardial infarction

## Abstract

A sensitive electrochemical immunosensor for the detection of the heart-type fatty acid binding protein (HFABP), an earlier biomarker for acute myocardial infarction than Troponins, is described. The sensing platform was enhanced with methylene blue (MB) redox coupled to carbon nanotubes (CNT) assembled on a polymer film of polythionine (PTh). For this strategy, monomers of thionine rich in amine groups were electrosynthesized by cyclic voltammetry on the immunosensor’s gold surface, forming an electroactive film with excellent electron transfer capacity. Stepwise sensor surface preparation was electrochemically characterized at each step and scanning electronic microscopy was carried out showing all the preparation steps. The assembled sensor platform combines MB and PTh in a synergism, allowing sensitive detection of the H-FABP in a linear response from 3.0 to 25.0 ng∙mL^−1^ with a limit of detection of 1.47 ng∙mL^−1^ HFABP that is similar to the clinical level range for diagnostics. H-FABP is a newer powerful biomarker for distinguishing between unstable angina and acute myocardial infarction.

## 1. Introduction

Cardiovascular diseases, including acute myocardial infarction (AMI), represent one of the most important health problems, being the main cause of death worldwide [1,2]. Obtaining a differential diagnosis between acute coronary syndrome (ACS) and non-ischemic chest pain is one of the major challenges in cardiac emergencies, especially in patients without change on the ST segment of the electrocardiogram (ECG), which is a classical profile for acute myocardial injury [3]. Although the ECG is the first recommended procedure for the detection of AMI, it does not have good diagnostic sensitivity, at approximately 50%, and therefore it cannot be exclusive for the diagnosis of AMI [4,5]. Several biochemical markers have demonstrated to be useful for the diagnosis of AMI for trial in cardiac emergencies, helping to quickly manage cases and enable an adequate treatment.

Cardiac biomarkers are components of cellular structures released into the circulation when a myocardial injury occurs. The quantification of the cardiac biomarkers in blood serum is relevant due to correlation of the information obtained with the severity and stage of cardiac damage [6,7,8]. Recently, the heart fatty acid binding protein (H-FABP) has been identified as an efficient early marker for myocardial ischemia and associated with cardiac troponins (cTnI and cTnT) for a more accurate diagnosis of AMI [9]. In a myocardial injury, H-FABP is readily released into the bloodstream within 20–30 min, being useful in the early diagnosis of AMI in a patient with unstable angina [10,11,12].

Given that H-FABP is an important biomarker for the management of cardiovascular injury, unstable angina, and AMI [13]; analytical tools for quantification of the HFABP in the bloodstream have been developed. In clinical practice, enzyme-linked immunosorbent assay (ELISA) and electro-chemiluminescence immunoassay (ECLIA) have been widely used. Despite these methods having good specificity and well-established sensitive responses, they are limited due to requiring sophisticated technical apparatus and a certain amount of time for analyses, as well as requiring specialized technical personnel [14,15]. Meanwhile, more practical and portable systems for H-FABP measurement at bedside have been developed using lateral flow tests based on chromatographic immunoassays [16]. Although more practical and faster than ELISA and ECLIA, these tests present only qualitative results, and a low sensitivity in general, implying a limited use [3]. Therefore, the development of rapid and quantitative methods that assist in an early diagnosis of AMI, aiming a quick intervention is imperative.

In the past decade, immunosensors based on electrical responses to antigen–antibody interactions have proved to be excellent rapid diagnosis tools due to their high sensitivity, practicality, and the possibility of real-time analysis [16]. Currently, the technology offered by electrochemical immunosensors has enabled the development of devices like point-of-care testing (POCT) that allow a practical diagnostic with a short time response, being at the moment the most attractive alternative for analytical tests [17]. Despite the great advancement of POCT during the COVID-19 pandemic, a global outbreak of coronavirus, challenges still exist in the use of POCT for the detection of analytes in low concentrations, as required for H-FABP or other biomarkers, being limiting factors for its wide usability [18,19].

An essential aspect for reaching a high sensitivity in the immunosensors is the biomolecules’ (antibodies or antigens) immobilization step. Aiming a maximal efficiency for an analytical response, the process of immobilization requires a correct amount of biomolecules immobilized, meanwhile preserving a high antigen–antibody binding capacity, which implies maximal charge and non-random orientation, and also major proximity to the electrode surface [20]. Carbon nanotubes (CNTs) have attractive physical properties due to their carboxyl groups, a high surface-to-volume ratio that is feasible for a great number of immobilized biomolecules, and a high conductivity that can lead to an increase in immunosensor sensitivity [21]. Different strategies can be used to anchor CNTs on the electrode surfaces; one of them is their combination with polymers [20]. Thionine is a phenothiazine dye with two amino groups symmetrically distributed on each side of its aromatic rings that can be linked together via –NH– bridges to form polythionine (PTh). PTh is a conductive polymer widely studied because of its electroactivity, since it exhibits a fast charge transfer capacity that enhances the sensor response, providing better control of the surface properties [22].

Label-free electrochemical immunosensors commonly carry out detection using voltammetric techniques, and their response is based on the reduction of the current suffered by the increase in the diffusion barrier on the surface of the electrode due to the antigen–antibody interaction, given the isolating nature of the biomolecules. The use of redox-active compounds immobilized on the surface of electrodes has been carried out with the aid of nanomaterials, such as carbon nanotubes [23], graphene [24], and others. Methylene blue (MB) is a cationic heterocyclic organic dye of the phenothiazine family that has been used for biosensing due to its excellent redox proprieties (−0.4 V e–0.1 V) [25], in addition to promoting more efficient electron transfer between the biomolecules and the electrode surface [26,27]. Here, the idea was to use the signal of MB oxidation to increase the diffusion current from the reaction with ferricyanide, increasing analytical efficiency. MB was linked to CNTs via sodium dodecyl sulfate (PSS), which is an anionic surfactant, working as a dispersing agent, as well as a binding agent for PTh due to its oppositely charged nature derived from sulfonic groups. Due to its catalytic activity, MB has been used for energy storage and capacitor applications [28]. Nevertheless, few studies have included MB incorporated into the sensing platform for electrochemical detection of immunoreactions using ferri/ferrocyanide. In the oxidation potential of [Fe(CN)_6_]^3−^/[Fe(CN)_6_]^4−^, approximately −0.4 V, electroactive species flow to the electrode surface, where methylene blue is immobilized. At this potential, uric acid, ascorbic acid, and other blood metabolites do not interfere in the generation of signal, since their oxidation is in positive potential. Thus, the use of MB as a chemical mediator in the immunosensor platform may allow greater analytical sensitivity due to the increase in generated current.

## 2. Materials and Methods

### 2.1. Reagents and Materials

Recombinant human H-FABP proteins and anti-H-FABP monoclonal antibodies were purchased from Abcam™ (Cambridge, UK). The standard calibrator serum samples were purchased from Sigma-Aldrich™ (St. Louis, MO, USA). Monomers of thionine acetate (Th), methylene blue (MB), sodium dodecyl sulfate (PSS), N-ethyl-N′-(3-dimethylamino propyl) carbodiimide, (EDC), N-Hydroxysuccinimide (NHS), 98% pure ethylenediamine (EDA), and sodium polysulfone (PSS) were also obtained from Sigma-Aldrich™ (St. Louis, MO, USA). Carbon nanotubes functionalized with carboxylic groups (CNT-COOH) 95% pure were obtained from Dropsens™ (Oviedo, Spain).

The saline phosphate buffer solution (PBS) (10 mM, pH 7.4) used in all experiments for the dilution of the serum samples was prepared by dissolving 0.2 g of KCl, 8.0 g of NaCl, 0.24 g of KH_2_PO_4,_ and 1.44 g of Na_2_HPO_4_ in 1000 mL of pure water obtained from a reverse osmosis unit (Human RO 180). Glycine was purchased from Fluka Analytical, H_2_SO_4,_ and potassium ferricyanide (K_3_[Fe(CN)_6_]) from Química Moderna, and potassium ferrocyanide (K_4_[Fe(CN)_6_]) from Vetec (Rio de Janeiro, Brazil).

The analytical response of the immunosensor was evaluated for proof of concept by subjecting the electrode of the H-FABP in incubations with serum sample calibrators. These standard calibrator serum samples used in this study were supplied by the Central Laboratory of Clinical Analysis of the Clinical Hospital from the Federal University of Pernambuco, Recife, Brazil; consisting of a gold standard for calibration and quality control of the immunoanalyzer, with H-FABP concentrations according to clinical ranges defined by suppliers. These calibrators are used for electrochemiluminescence immunoanalyzer equipment. In this study, different concentrations of H-FABP were PBS-diluted.

### 2.2. Electrochemical Measurements and Equipment

Electrochemical measurements were performed using the Autolab PGSTAT204 potentiostat (Eco Chemie, Utrecht, The Netherlands), controlled by the Nova 2.1.3^®^ data acquisition and processing software. All electrochemical measurements were performed using a conventional three-electrode system, composed of the conventional polycrystalline gold electrode (GE) with a circular section of 0.8 mm diameter as the working electrode, Ag/AgCl electrode (KCl sat) as reference electrode, and a helical platinum wire as a counter electrode in a 5 mL electrochemical cell. All electrochemical measurements were performed in the presence of a redox probe, 5 mM (K_3_[Fe(CN)_6_])/(K_4_[Fe(CN)_6_]) solution.

For electrosynthesis of the polythionine film (PTh) on the gold electrode surface (GE), the cyclic voltammetry technique was used with a scan rate of 100 mV∙s^−1^ in a potential window between −0.5 to 0.9 V for 30 scans. The analytical responses and the electrochemical characterizations of the steps for assembling the sensor platform were obtained by using the cyclic voltammetry (CV) technique in a potential window of −0.8 V to 0.8 V, at 30 mV∙s^−1^ scan rate. The data for analysis and analytical responses were processed in the Origin Lab™ software, version 8.0.

### 2.3. PTh Film and Nanostructured Platform Preparation

Before modifications, the GE surface was subjected to a cleaning procedure performed by polishing in alumina paste (0.3 µm), making movements in the form of infinity (∞) for 2 min. To verify the removal of residues from the electrode surface, cyclic voltammograms were recorded in a 5 mM (K_3_[Fe(CN)_6_])/(K_4_[Fe(CN)_6_]) redox probe, in which the differences between anodic and cathodic potentials (Epa and Epc, respectively) were less than 90 mV.

After cleaning, the GE was immersed in an electrochemical cell containing a solution of 0.1 mM of thionine monomers prepared in 10 mM PBS (pH 7.4), and subjected to electrosynthesis by the cyclic voltammetry technique. Subsequently, the nanostructured film was obtained by drop-casting a solution containing carboxylated CNTs functionalized with MB (CNT@MB) previously prepared. 

To prepare the CNT@MB solution, 1 mg of carboxylated CNTs was dispersed in an aqueous solution of PSS (1 mg∙mL^−1^) and subjected to an ultrasonic bath for 2 h, followed by incubation at 50 °C, overnight [29]. Afterward, the solution containing the CNTs PSS activated was centrifuged at 15,000 RPM for 45 min and the CNTs were suspended in 1 mL of 10 mM MB [30]. CNT@MB composite was subjected to the ultrasonic bath for 2 h and exhaustively washed using a centrifuge at 15,000 RPM until the supernatant was colorless, indicating that the excess of unbound MB was removed. 

### 2.4. The Anti-H-FABP Immobilization and Blocking of Non-Specific Bindings

After the electrode assembly (CNT@MB/PTh/GE), the immobilization of anti-H-FABP antibodies was performed. To promote an oriented immobilization of antibodies, a mixture of 20 mM EDC and 50 mM NHS [1:1] in PBS buffer (pH 7.4) was left to react with the electrode at room temperature (~23 °C) for 40 min to activate carboxylic groups present in the CNTs. Simultaneously, a mixture containing 5 µL of EDA (20%) and 10 µL of anti-H-FABP (20 µg∙mL^−1^) was left to react for 40 min and then deposited on the electrode for 60 min for immobilization. Afterward, the electrode was thoroughly washed in water to remove the unbound excess material. The non-specific sites on the electrode surface were blocked with a glycine solution (100 mM) prepared in 0.01 M PBS (pH 7.4) by incubating for 30 min. After washing with ultrapure water, the prepared electrode consisted of a reactive H-FABP surface (Glycine/Anti-H-FABP/CNT@MB/PTh/GE).

### 2.5. Analytical Responses to the H-FABP

**Figure 1:**
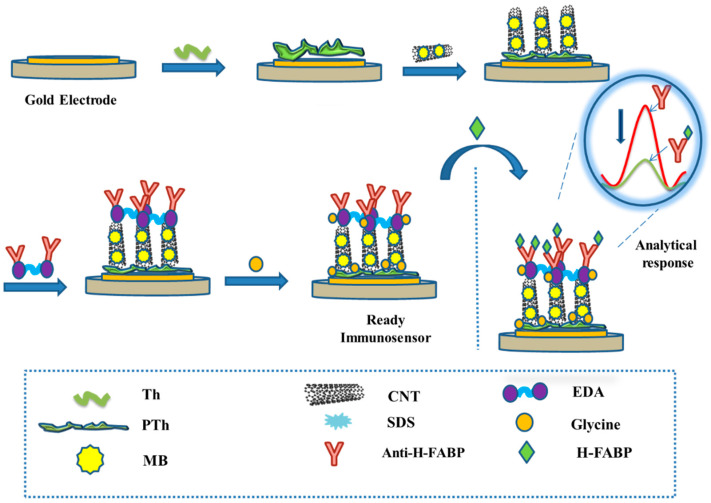
Stepwise step modifications of the electrode surface for immunosensor assembling, and immunoassay for analytical responses.

The immunosensor was subjected to the H-FABP samples in different concentrations by incubating for 30 min. Before readings, the electrode surface was washed with PBS (pH 7.4) to remove non-specific bindings. All steps of the assembling of the immunosensor and the immunoassay are outlined in Figure 1.

## 3. Results and Discussion

### 3.1. PTh Film Electrosynthesis

**Figure 2:**
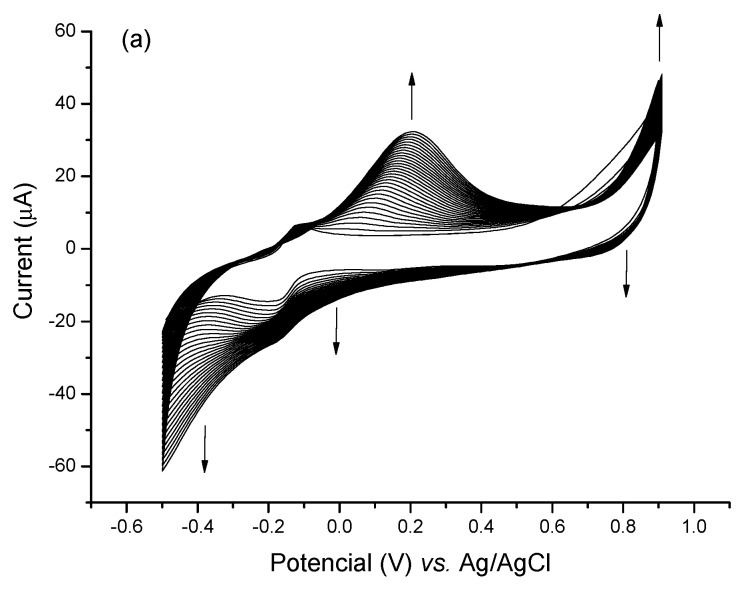

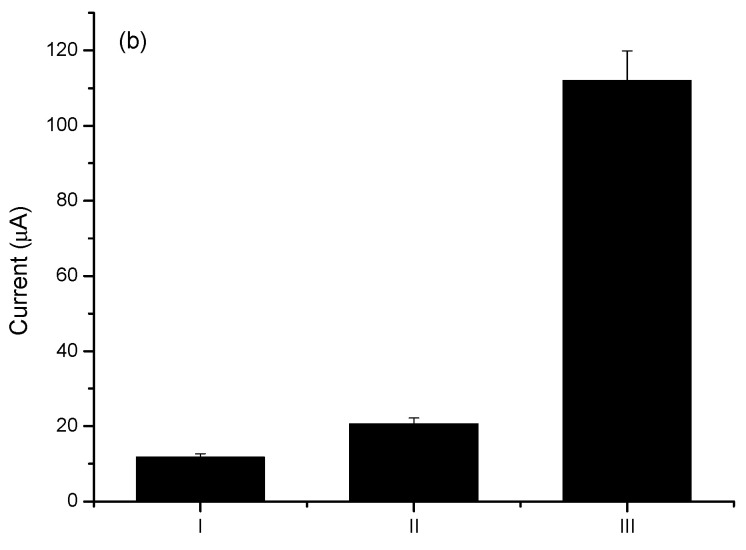
(**a**) Cyclic voltammograms of PTh electropolymerization process for 30 successive cycles in the presence of PBS (7.4) as support electrolyte. (**b**) Bar diagram indicating standardized electroactive areas to the clean electrode in the respective assembly steps (I) Bare GE, (II) PTh/GE, (III) CNT@MB/PTh/GE. The experiment was carried out in the presence of 0.1 M KCl as an electrolyte.

The electropolymerization performed in a solution of thionine monomers yielded PTh films tightly adhered to the sensor surface due to the presence of sulfur moieties attached to the aromatic ring of the molecule, thus favoring its adhesion to the gold surface [30]. Also, PTh polymeric thin films have the advantage of introducing highly stable, electroactive, and efficient redox centers to sensor surfaces. Electrosynthesis of the PTh was carried out by CV technique, and the formation of the polymer was confirmed by the increase in the anodic and cathodic peaks by 0.2 and −0.2 V, respectively (Figure 2a). An increase in the reduction current between the −0.4 V and −0.5 V potential was also observed, indicating that both lateral amino groups of the thionine monomers were involved in the polymerization.

Thionine monomers synthesized in alkaline media result in less conductive polymers. Owino et al. (2008) obtained thionine films of a multiporous nature with a high yield when electropolymerization was carried out at pH 6.5 in an acetate buffer (0.1 M). In this study, electropolymerization started from the oxidation of the thionine –NH_2_ groups in potential below 1.1 V vs. Ag/AgCl [22]. This high yield can be attributed to thionine’s high pKa of 11.3. Here, the PTh films were electrosynthesized in PBS buffer (pH 7.4), and the addition of the CNTs resulted in a 75% increase in the average electroactive area. 

### 3.2. Assembly of the CNT@MB Nanostructures on PTh Films

It is well known that the use of CNTs in sensor platforms improves analytical responses due to their large electrical conductivity that involves their electronic and morphological aspects, attributed to the presence of intercalated sp^2^ bonds that increase the electric transfer rate [31,32]. Although there was a decrease in conductivity due to the decrease in the CV area, the introduction of PSS resulted in greater stability in the bond with the PTh film.

The PTh film formed on the electrode surface had a cationic characteristic [33] that favors easy binding of the CNTs dispersed in PSS through electrostatic interactions by sulphonic groups [32].

**Figure 3:**
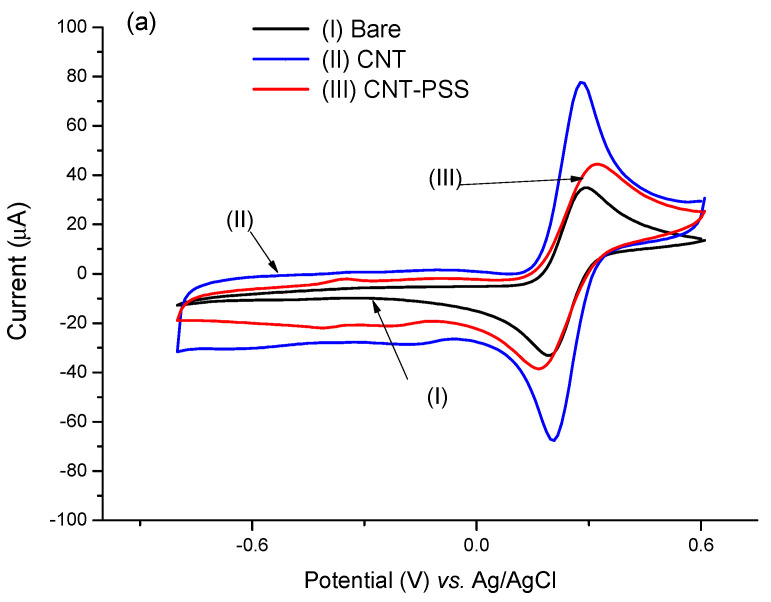

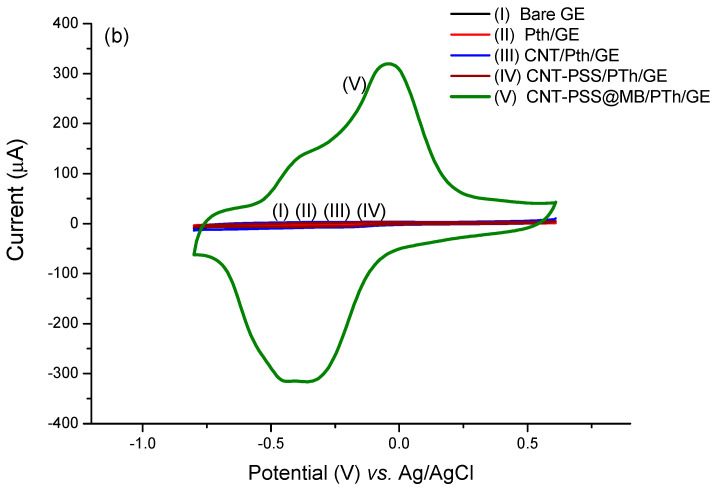
Voltammetric profiles performed in the CNT@MB/PTh/GE. (**a**) Effect of PSS on CNT. (**b**) Effect of CNT-PSS@MB on the assembling of the electrode.

Herein, the increase in conductivity resulting from the addition of CNTs to the platform was confirmed by the significant increase in the electroactive area when compared to the electrode with PTh film only (Figure 3a). The estimative was based on the Randles-Sevick equation (Equation (1)):

(1) $$I_{p}=\left(2.69\times 10^{5}\right)\;{A\;D}^{\frac{1}{2}}\;n^{\frac{3}{2}}{\;\nu }^{\frac{1}{2}}\;C$$

where Ip is the peak current value, A represents the electroactive area of the electrode (cm^2^), D is the diffusion coefficient of the solution (cm^2^∙s^−1^), n is the number of electrons involved in the redox reaction, ν is the potential scan rate (V∙s^−1^), and C is the concentration of the probe molecule in solution [34].

The way that CNTs are attached to detector surfaces can affect the analytical responses of the immunosensor [30]. Different polymers have been used as anchors for binding CNTs, especially those that present –COOH, –SH, and –NH_2_ functional groups in their structures [35]. Good stability was evidenced, attributed to strong linkage of PTh to the electrode surface and of PTh to the CNTs (Figure S1). The PTh polymer containing –NH_2_ with cationic characteristics had the advantage of obtaining electrostatic interactions with PSS or with carboxylic groups of the CNTs [36]. Thionine is a small planar molecule with a phenothiazine ring with two amino groups symmetrically distributed on each side, which can interact with CNTs [37,38]. 

**Figure 4:**
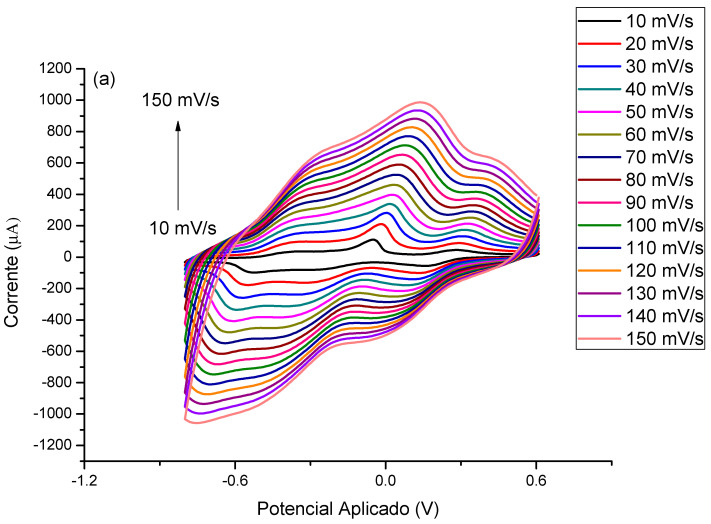

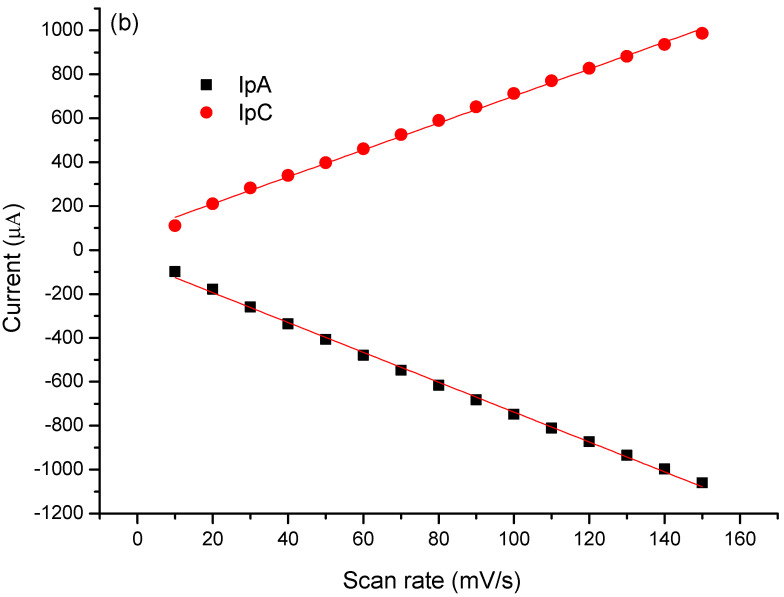

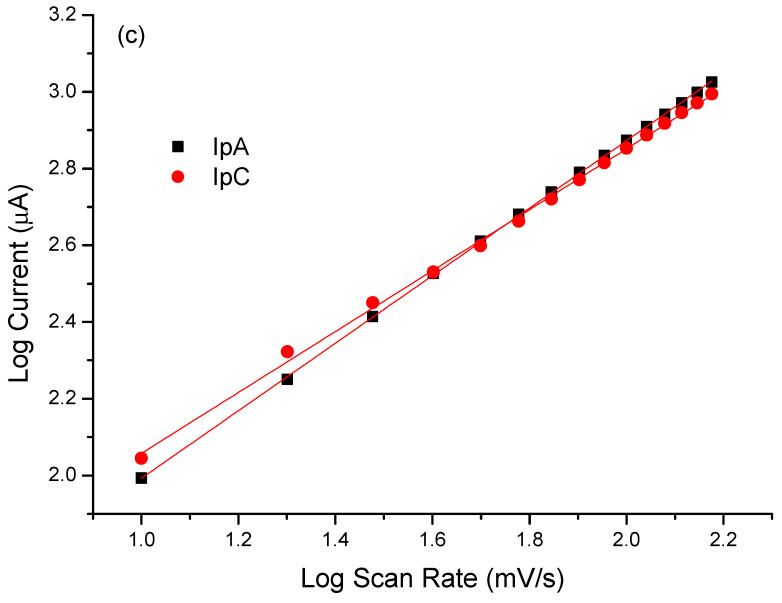
(**a**) Voltammetric profiles of the EAu/PTh/CNT@MB surface obtained under different scan rates. (**b**) Plot of scan rate vs. anodic and cathodic peaks. (**c**) Log of scan rate vs. log of the modulus of the anodic and cathodic peaks. Experiments were performed in the presence of PBS (pH 7.4).

In order to better evaluate the contribution of MB to the immunosensor response and its electrocatalytic role, cyclic voltammograms in the absence of a redox probe solution were performed. The modified electrode with PTh/CNT@MB was subjected to different scan rates in the presence of PBS (pH 7.4) (Figure 4a). According to Figure 4b, a linear increase of anodic and cathodic current peaks with the increase of scan rate varying from 10 to 150 mV∙s^−1^. These redox peaks were attributed to the MB immobilized on the electrode. After plotting was abserved the current of anodic and cathodic peaks as a function of scan rate, a straight lines was observed, exhibiting correlation coefficients of 0.989 and 0.987, respectively, proving the MB was successfully immobilized on the electrode surface [35] (Figure 4b). Deeper study analyzing the plot of slope of the log of scan rate vs. the log of modulus of the anodic and cathodic peaks showed 0.81 and 1.10, respectively, indicating an adsorptive process but with a discrete electron-diffusion, which means a mixed process since the slope approximately 0.75 (Figure 4c) [23]. Thus, electroactive species of the MB are confined on the electrode surface and simultaneously are led to the bulk in a diffusion-controlled process.

Stability studies of the CNT@MB/PTh/GE were carried out by subjecting the electrodes to 20 consecutive cycles of cyclic voltammetry (50 V∙s^−1^, potential window between −0.8 to 0.8 V). The coefficients of variation of redox current peaks of the CVs calculated demonstrated stability and operational reproducibility of the platform. The repetition of a redox peak pattern was observed in the successive voltammetric measurements of the modified electrode, with no significant displacement of the anodic (Ipa) and cathodic (Ipc) current redox peak potentials (Figure S1). The coefficients of variation calculated for Ipa and Ipc were approximately 3% for both. This good stability can be attributed to the strong bindings already described between the PTh film and CNTs [28]. 

Figure 3b demonstrates the catalytic contribution of the MB with a dramatic increase in electron transfer. The catalytic cycle of MB and ferricyanide/PSS is well known [39]. Upon reduction of MB to leucomethylene blue, it is capable of reducing ferro/ferricyanide freely diffusing in solution. The leucomethylene blue is then reoxidized to methylene blue. The ferro/ferricyanide acts as a diffusing electron sink in solution for the MB that transfers the electroactive species to the electrode. A dramatic increase (approximately 770%) in the electroactive area was observed in relation to the electrode with the PTh film, according to the Randles–Sevcik equation (Figure 3b).

Scan rate studies were carried out to evaluate the adsorption of MB in the modified CNT@MB/PTh/GE nanocomposite electrode in the presence of the redox probe 5 mM (K_3_[Fe(CN)_6_])/(K_4_[Fe(CN)_6_]) in 0.1 KCl in the potential window between −0.8 to 0.8 V with scan rates ranging from 10 to 150 mV∙s^−1^ (Figure S2a). A linear increase was observed in anodic and cathodic peaks (Ipa and Ipc, respectively) with the square root of scan rate (Figure S2b), showing an adjusted r for Ipa = 0.997 and Ipc = 0.992, suggesting a linear regression that means a diffusion-controlled process. [Fe(CN)_6_]^3−^/[Fe(CN)_6_]^4−^ is a well-studied redox couple, which can participate in the faradaic current by a gradient concentration of redox species in the bulk solution (Figure 4c). After a deeper analysis of the plot of the log scan rate vs. log anodic and cathodic peaks (in modulus), a mixer process was observed, since the linear coefficient slopes were 0.87 and 0.88 for anodic and cathodic peaks, respectively [23]; there are currents resulting from diffusion of redox species in the solution, as well as faradaic current from species by migration due to changes in the double layer on the electrode surface, which was greatly increased by the presence of MB@CNT.

#### Morphological Characterization

**Figure 5:**
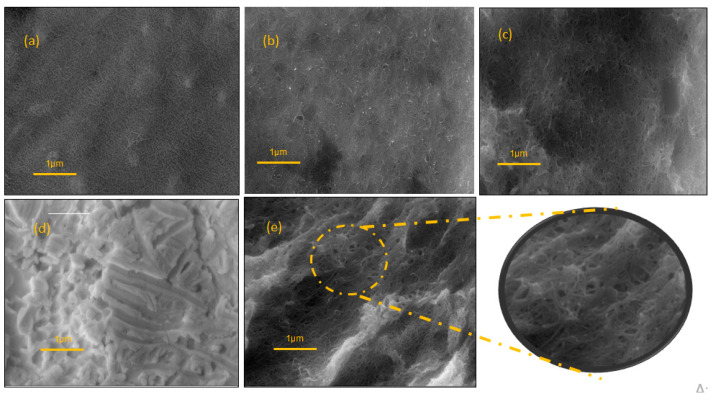
SEM images (**a**) CNT/GE (**b**) PSS-CNT/GE; (**c**) PSS-CNT@MB/GE; (**d**) PTh/GE; (**e**) PSS-CNT@MB/PTh/GE. Mag 16.10 KX.

Scanning electron micrography was performed to study the conformational structures of the (a) CNT/GE; (b) PSS-CNT/GE; (c) PSS-CNT@MB/GE; (d) PTh/GE; (e) PSS-CNT@MB/PTh/GE. Figure 5a shows the presence of CNTs evidenced by the characteristic spaghetti-like tubular nanostructures, which are typical of CNTs. PSS has been reported as a good dispersant and dopant for CNTs, in micrograph 5b can be observed the nanostructures embedded in PSS, showing an efficient dispersion. PTh is an electroactive film with a high porosity, which is possibly a more irregular surface with larger pores compared with the image obtained with CNT-PSS 5c. It is also possible to observe that the presence of the PTh generated a more irregular surface, with a consequent increase in the surface area compared with the electrode without PTh. After MB incorporating into CNT-PSS, an increase in porous surface formation induced by PTh and white points in the sensor surface were observed 5e; the exploded view shows CNTs homogeneously embedded in the dispersion with large pores due to the presence of PTh. The synergic effect obtained with MB@CNT and PTh is clearly explained by the increase in the cyclic voltammogram area, as shown in Figure 3b. In the SEM micrographs, CNTs are commonly described presenting spaghetti-like images, which are not observed in Figure 5d, showing the PTh polymer with the SEM images more difusses (Figure 5c,e), due to a more significant scattering of electrons, demonstrating high capacity of CNTs for electron transfer, as was also evidenced by cyclic voltammetry.

### 3.3. Anti-H-FABP Antibodies Immobilization

The immobilization of Anti-H-FABP antibodies was performed considering the reactive amino groups present in the bi-functional agent (EDA) that allowed covalent immobilization with the carboxylated sites present both in the Fc portion of the antibodies and the CNTs, thus generating greater stability for the platform. For this procedure, the Fc terminal of the anti-H-FABP antibodies was previously activated.

The immobilization of the antibody was confirmed by a reduction in the voltammogram current peaks which indicated the presence of Anti-H-FABP on the sensor platform [33]. A glycine solution was used to block non-specific bonds, which also caused a reduction in the current peak [31].

In exploratory studies to optimize the concentration of antibodies used in the anti-H-FABP electrode, an initial concentration was established as a function of the anodic current responses (Figure S4a). From statistical analysis, significant exponential behavior was confirmed by the increased concentrations of immobilized antibodies (*p* < 0.05, Chi-square test, n = 6). The optimal concentration was found at 20 µg∙mL^−1^ of anti-H-FABP, as a plateau (Figure S4b), according to Equation (2):

Ipa = I_0_ [anti-H-FABP](initial) + Ae^−x/t1^ (2)

where Ipa represents the anode peak current, I_0_ [anti-H-FABP] (initial) is the starting antibody concentration, A is the dynamic component, x is the concentration of antibodies at desired time, t1 is the exponential growth constant of the concentration of anti-H-FABP antibodies.

### 3.4. Analytical Response to the H-FABP

**Figure 6:**
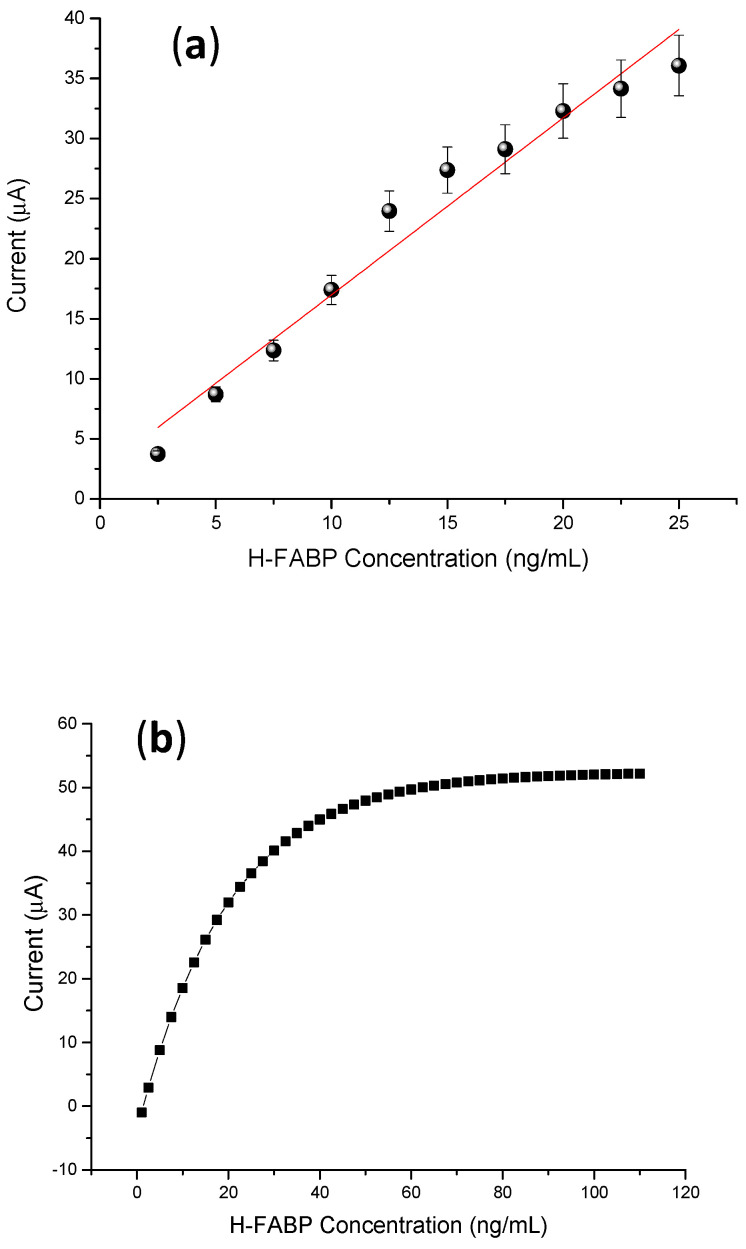

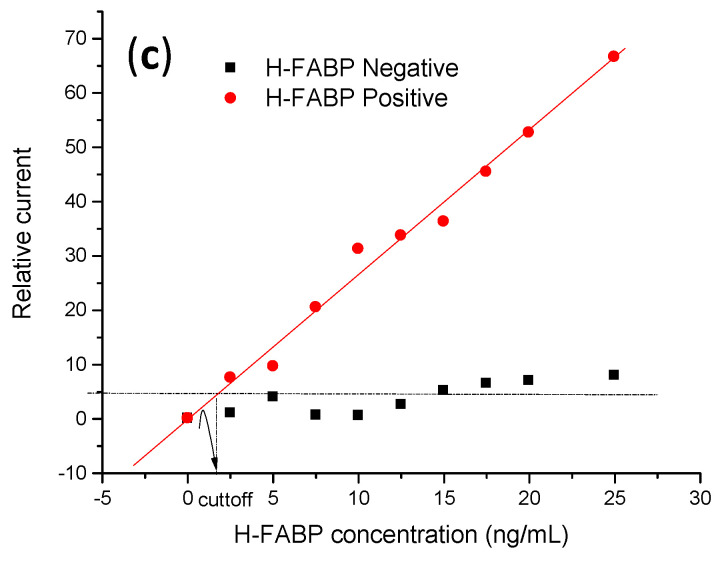
(**a**) Calibration curves of different concentrations of H-FABP (2.5; 5; 7.5; 10; 12.5; 15; 17.7; 20; 22.5; 25 ng∙mL^−1^). Measurements were obtained in the presence of 5 mM of (K_3_[Fe(CN)_6_])/(K_4_[Fe(CN)_6_]), at 30 mV∙s^−1^ scan rate. (**b**) Linear adjustment of the calibration curve. (**c**) Negative and positive H-FABP concentrations in standard calibrator serum samples, in the presence of 5 mM of (K_3_[Fe(CN)_6_])/(K_4_[Fe(CN)_6_]) at mV∙s^−1^ scan rate. In (**a**) the mean and standard deviation were obtained from testing three samples.

A linear increase in the anodic peaks was observed with the increase in the H-FABP concentrations present in the samples prepared in PBS. A linear range was established between 2.5 to 25 ng∙mL^−1^ of H-FABP (Figure 6a), being met in clinical range levels [40,41]. This test proves superior to lateral flow tests, which recognize fatty acid protein antigens only between 7 to 10 ng∙mL^−1^ [42]. In addition, the system had good analytical sensitivity (limit of detection—LOD = 1.47 ng∙mL^−1^) and good reproducibility, with a coefficient of variation of 6.1% (n = 10 tests), showing better results than commercial chromatographic tests (<10%) [42]. The analytical curve was validated by theoretical studies, confirming a linear range up to 25 ng∙mL^−1^ H-FABP (Figure 6c).

As proof of concept for analytical responses, the specificity was evaluated by subjecting the immunosensor to successive incubations with gold standard positive H-FABP and negative (control) calibrator serum samples supplied by clinical service. According to the curve in Figure 6c, this H-FABP immunosensor exhibited a linear response with a broad range between 2.5 to 25 ng∙mL^−1^. The limit of discrimination, in this case, was higher than samples prepared in PBS due to interferents that bind to the electrode surface, promoting a diffusion barrier on the electrode surface and reducing the current response. The estimated limit of detection was 1.7 ± 0.2 ng∙mL^−1^, calculated according to IUPAC [43]. Herein, the linear range for clinical diagnosis found in this study was also superior to the limits obtained with lateral flow devices [44]. For selectivity, the real discriminatory limit was obtained by cutoff, which means that up to this value, the immunosensor was able to distinguish H-FABP from non-specifical bindings. In this study, the cutoff value was established by average responses of the anodic current from the CVs from negative sera (control) plus 20%, obtained from linear curve (2.5–25.0 ng∙mL^−1^), CI = 95%, and Pearson r 0.977), established as statistically significant (*p* < 0.05) It was confirmed that the immunosensor showed high differentiation to discriminate H-FABP and interferents, with the advantage of not needing a second labeled antibody to increase the diagnostic specificity, a limitation that is necessary for enzyme immunoassay tests [45,46]

Considering that clinical levels of HFABP are between 1.6 ng∙mL^−1^ to 19 ng∙mL^−1^ in various studies of cardiovascular disease [45], this immunosensor presented a clinical range compatible with the biomarker levels. Many biosensors for H-FABP have been developed in the past decade, motivated by the importance of determining unstable angina and acute myocardial infarction. Stan et al. (2012) developed an impedimetric immunosensor with limits of detection lower than 1 ng/mL (0.9 ng∙mL^−1^), but with a range superior similar to our study, furthermore using an impedance method for detection which is much more complex [47]. Karaman et al. (2021) employed nanoparticles and quantum dots to reach a more sensitive limit of detection (LOD) (3.30 fg∙mL^−1^) with an apparatus of a sandwich-like system similar to an ELISA, employing antibody-conjugated nanoparticles [48]. Here, the effect of methylene blue (MB) redox coupled to carbon nanotubes (CNTs) assembled on a polymer film of the polythionine (PTh) promoted a synergism, resulting in a sensitive and simple system to acquire label-free measurements.

## 4. Conclusions

A label-free electrochemical sensing platform composed of a PTh polymer film and nanomaterials conjugated to the MB mediator to increase the redox activity derived from ferri/ferrocyanide was developed, indicating good prospects for the development of a label-free immunosensor for the high-sensitivity detection of anti-H-FABP antibodies. Analytical results, mainly LOD and cut-off, met the desirable range of clinical levels relevant for routine clinical cardiac diagnosis.

## Data Availability

Not applicable.

## Supplementary Materials

The following supporting information can be downloaded at: https://www.mdpi.com/article/10.3390/bios13090873/s1, Figure S1. Electrochemical stability study of the film showing Ipa and Ipc responses of CVs performed in 20 successive cycles in the presence of supporting electrolyte (0.1 M KCl). Figure S2. Voltammetric profiles of (a) EAu/PTh/CNT and (b) EAu/PTh/CNT/PSS performed in the presence of 5mM of (K_3_[Fe(CN)_6_])/(K_4_[Fe(CN)_6_]), at a scan rate of 30 mV∙s^−1^. Figure S3. (a) Voltammetric profiles of the EAu/PTh/CNT@MB surface obtained under different scan rates; (b) Plot of the square root of the scan rate vs. anodic and cathodic peaks. Experiments performed in the presence of 5mM of (K_3_[Fe(CN)_6_])/(K_4_[Fe(CN)_6_]). Figure S4. (a) Curve of the optimization the concentration of the anti-HFABP obtained from cathodic peaks. (b) Theoretical curve from experimental data in (a).
