# Supplementary material for: A Methylene Blue-Enhanced Nanostructured Electrochemical Immunosensor for H-FABP Myocardial Injury Biomarker
Source: Biosensors (Basel). 2023 Sep 7;13(9):873. doi: 10.3390/bios13090873 (PMC10526172; doi:10.3390/bios13090873)
Supplement: Supplementary file 1 [file biosensors-13-00873-s001.zip › biosensors-2556625-supplementary.pdf]

biosensors

MDPI

Supplementary Materials

A Methylene Blue-Enhanced Nanostructured Electrochemical Immunosensor for H-FABP Myocardial Injury Biomarker

Cecília Maciel Prado, Paula A. B. Ferreira, Lucas A. Lima, Erika K. G. Trindade and Rosa F. Dutra *

CAPTIONS

Figure S1. Electrochemical stability study of the film showing Ipa and Ipc responses of CVs performed in 20 successive cycles in the presence of supporting electrolyte (0.1 M KCl).

Figure S2. Voltammetric profiles of (a) EAu/PTh/CNT and (b) EAu/PTh/CNT/PSS performed in the presence of 5mM of ($K_3[Fe(CN)_6]$)/($K_4[Fe(CN)_6]$), at a scan rate of 30 mV s.

Figure S3. (a) Voltammetric profiles of the EAu/PTh/CNT@MB surface obtained under different scan rates; (b) Plot of the square root of the scan rate vs. anodic and cathodic peaks. Experiments performed in the presence of 5mM of ($K_3[Fe(CN)_6]$)/($K_4[Fe(CN)_6]$).

Figure S4. (a) Curve of the optimization the concentration of the anti-HFABP obtained from cathodic peaks. (b) Theoretical curve from experimental data in (a).

Figure S1.

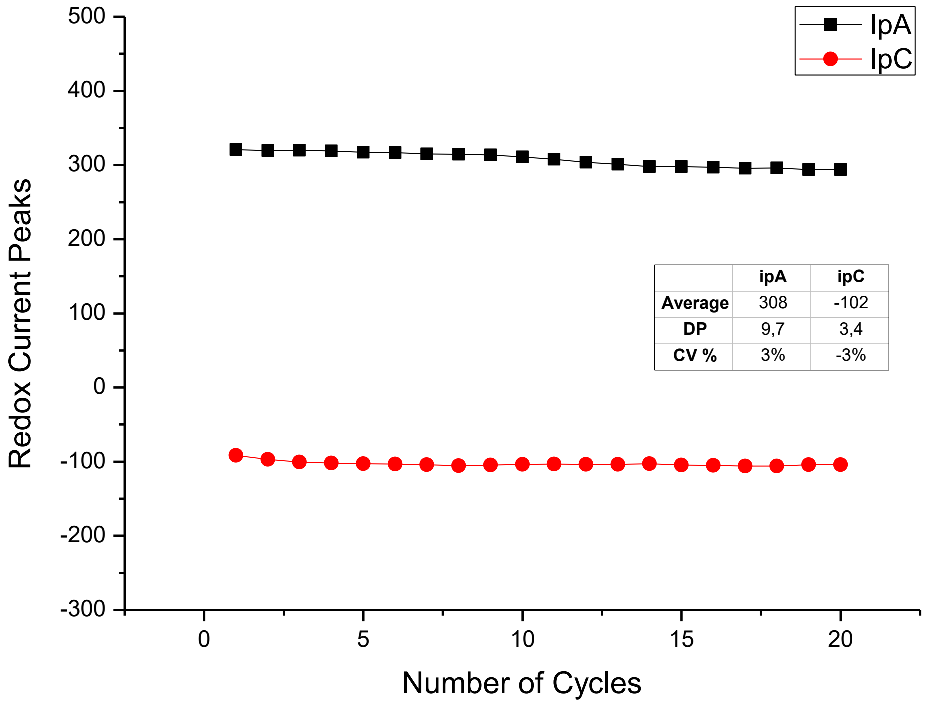

Figure S1. Electrochemical stability study of the film showing Ipa and Ipc responses of CVs performed in 20 successive cycles in the presence of supporting electrolyte (0.1 M KCl).

Figure S2.

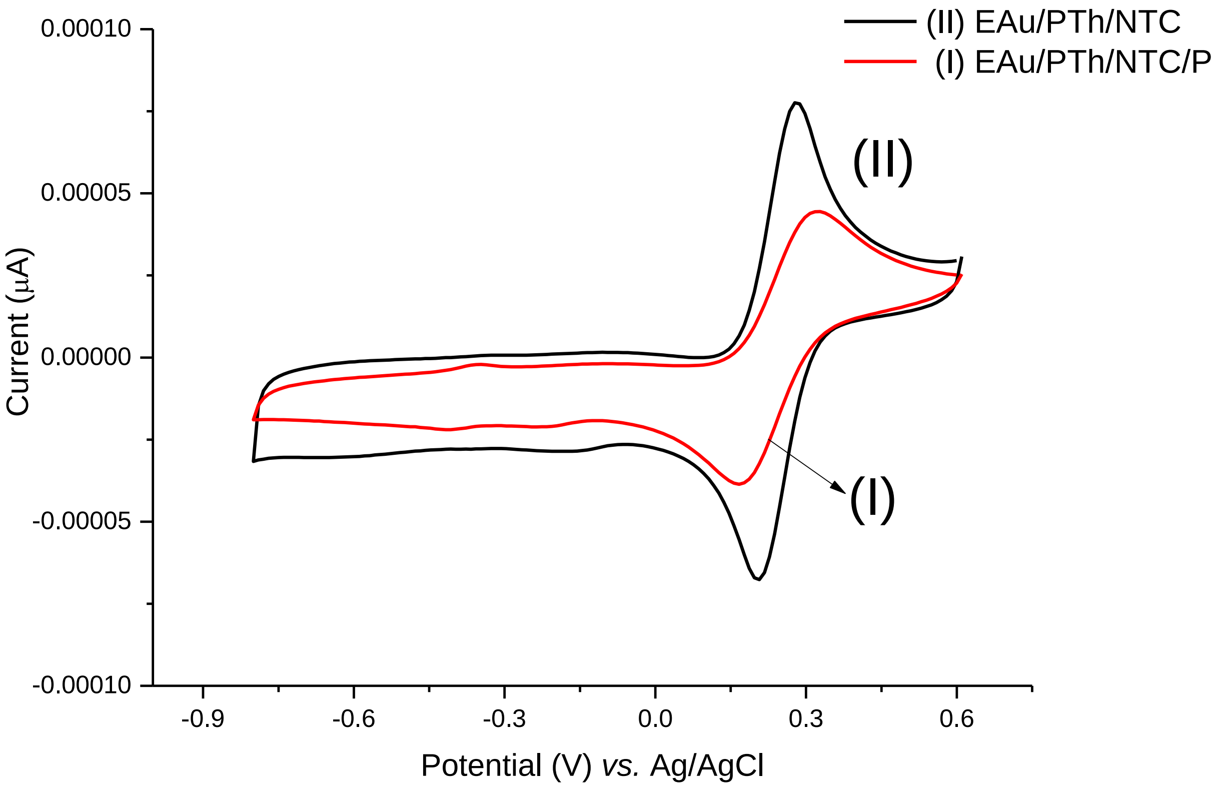

Figure S2. Voltammetric profiles of (a) EAu/PTh/CNT and (b) EAu/PTh/CNT/PSS performed in the presence of 5mM of ($K_3[Fe(CN)_6]$)/($K_4[Fe(CN)_6]$), at a scan rate of 30 mV s.

Figure S3.

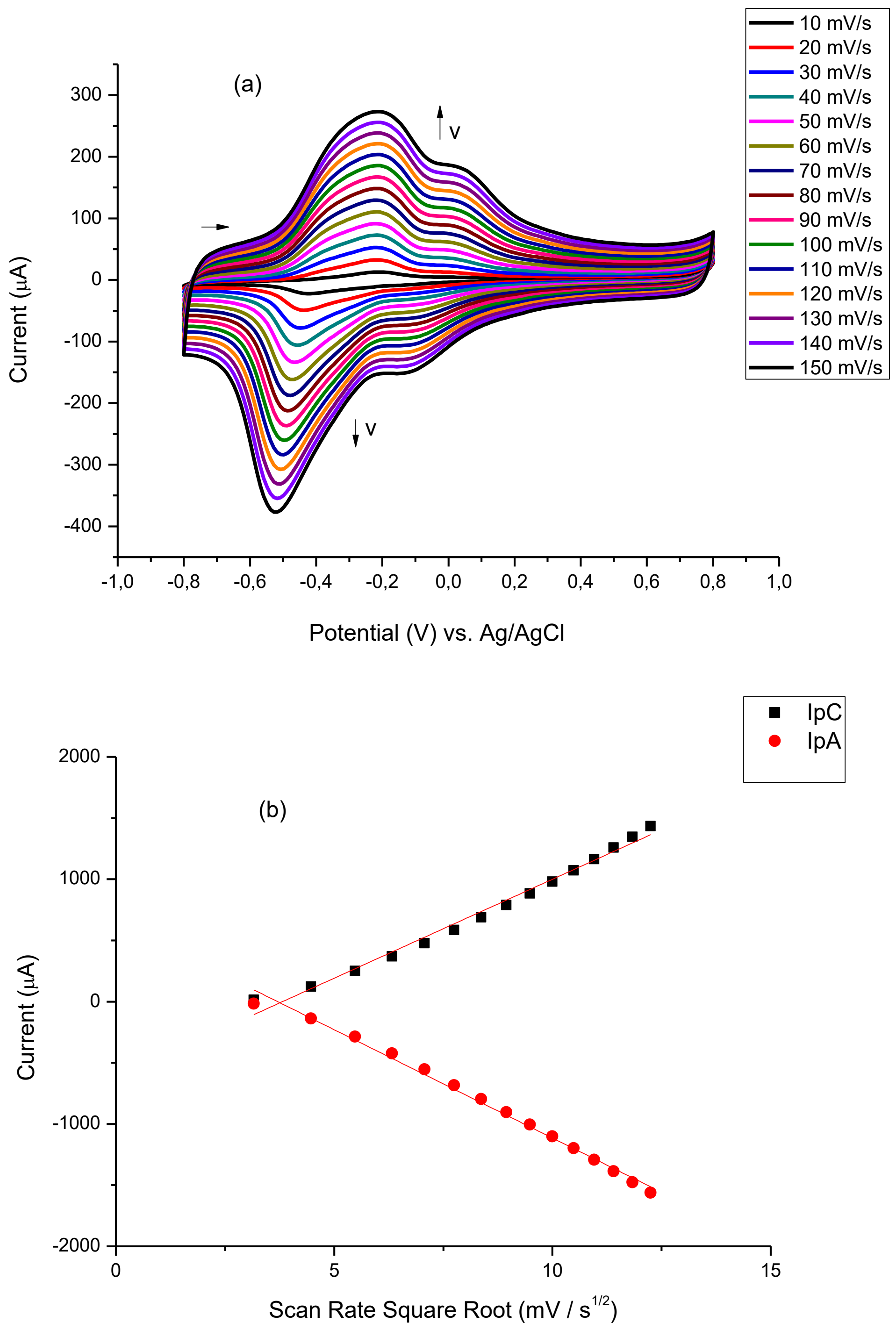

Figure S3. (a) Voltammetric profiles of the EAu/PTh/CNT@MB surface obtained under different scan rates; (b) Plot of the square root of the scan rate vs. anodic and cathodic peaks. Experiments performed in the presence of 5mM of ($K_3[Fe(CN)_6]$)/($K_4[Fe(CN)_6]$).

Figure S4.

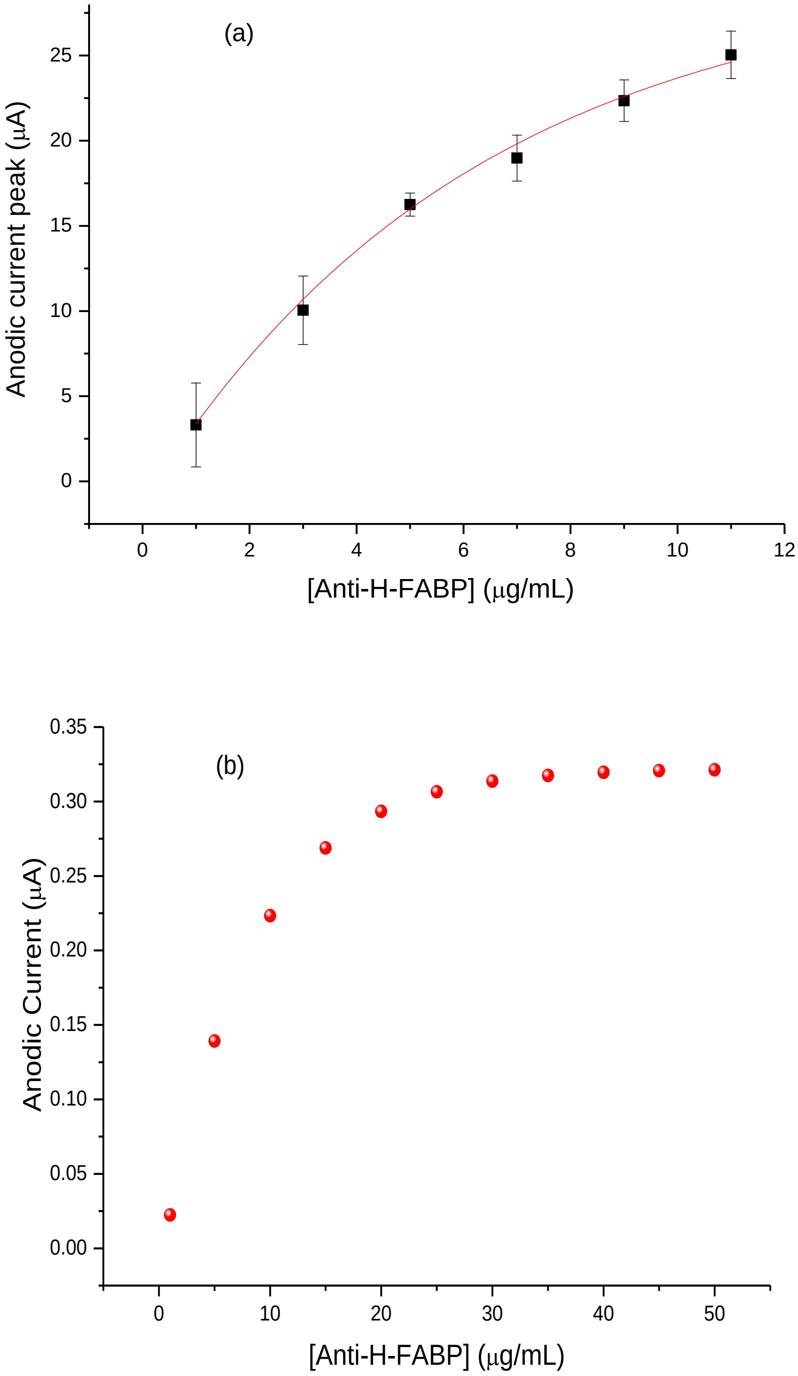

Figure S4. (a) Curve of the optimization the concentration of the anti-HFABP obtained from cathodic peaks. (b) Theoretical curve from experimental data in (a).
